# Greater than the Sum: Applying Daily-Dose Equivalents to Antipsychotic Prescription Claims to Study Real-World Effects

**DOI:** 10.3389/fphar.2021.709349

**Published:** 2021-08-06

**Authors:** Kaleen N. Hayes, Tara Gomes, Mina Tadrous

**Affiliations:** ^1^ Dalla Lana School of Public Health, Department of Public Health Sciences, University of Toronto, Toronto, ON, Canada; ^2^ Dalla Lana School of Public Health, Institute of Health Policy, Management, and Evaluation, University of Toronto, Toronto, ON, Canada; ^3^ ICES, Toronto, ON, Canada; ^4^ Li Ka Shing Knowledge Institute, Toronto, ON, Canada; ^5^ Leslie Dan Faculty of Pharmacy, Department of Pharmaceutical Sciences, University of Toronto, Toronto, ON, Canada; ^6^ Women’s College Hospital, Toronto, ON, Canada

**Keywords:** antipsychotic agents, dementia, dose-response relationship, drug, drug prescriptions, pharmacoepidemiology/methods

## Abstract

Traditional methods to standardize exposures in pharmacoepidemiologic studies, like defined daily-doses, may be inadequate to capture drug class effects when there are many in-class medications, formulations, and administration routes. Antipsychotic medications are one example of a drug class with these complexities. Direct dose conversion methods are pharmacologically-based but often overlooked, potentially for lack of real-world guidance and examples of their implementation. The purpose of this article is to describe a method to implement dose conversion, using an example study that quantifies antipsychotic use among a cohort of older adults with dementia. We identified 45,442 older adults (aged ≥66 years) with dementia initiating antipsychotic therapy between January 1, 2009 and December 31, 2012 in Ontario, Canada using linked administrative healthcare databases. We developed and applied a data cleaning and dose conversion algorithm to quantify antipsychotic exposure in chlorpromazine dose equivalents at initiation, month 6, and month 12 of therapy. Results were stratified by route of administration. At initiation, 14% of patients received multiple antipsychotic prescriptions simultaneously. Patients initiating regular injectable and multiple administration routes received the highest median chlorpromazine equivalent daily-doses. Data cleaning changed 3, 16, 36, and 42% of total equivalent daily-doses in patients initiating oral, regular injectable, long-acting injectable, and multiple administration routes, respectively. Dose conversion of prescription claims data was a feasible method to quantify and present antipsychotic drug exposures. Dose conversion methods can be considered for drug effects studies of antipsychotic therapies and other medication classes with complex use.

## Introduction

A challenge in pharmacoepidemiology is the ascertainment of drug exposures from prescription claims. Exposure assessment is further complicated when studying effects of an entire drug class, as some standardization, combination, or other manipulation of in-class exposure measurements is required. The following criteria pose particular difficulty for exposure assessment within a drug class: 1) the class contains many drugs, 2) in-class agents are available in multiple dosage forms and routes of administration, and 3) patients may be exposed simultaneously to multiple drugs within the class. Antipsychotics, benzodiazepines, and opioids are examples of drug classes with these complexities.

To illustrate the challenges of summarizing drug-class exposures, we present antipsychotic medications as an example. The use of antipsychotics to treat behavioural and psychological symptoms of dementia (BPSD) is associated with an increased risk of mortality and morbidity (Gill et al., 2007). Thus, quantifying and understanding the use and dosing of antipsychotic therapies in this population is paramount (Schneider et al., 2006; Gill et al., 2007) Although antipsychotics have myriad pharmacologic mechanisms of action (Sultana et al., 2017), a commonality is that all antipsychotics demonstrate dopamine receptor antagonism (Siafis et al., 2018). However, there are 67 different antipsychotics listed in the Anatomical Therapeutic Chemical (ATC) index, with many administration routes (e.g., injection and oral) and formulations (e.g., short-versus long-acting) (World Health Organization, 2019). Further, antipsychotics may be combined as dual therapy or long-acting maintenance therapy plus “as needed” doses (Tadrous et al., 2015; Ortiz-Orendain et al., 2017). Because of these complexities, it becomes difficult to understand the true burden of a population’s antipsychotic exposure, appropriateness of dosing, and additive effects of combination therapy.

To avoid these challenges, studies may exclude patients exposed to multiple antipsychotics (Tadrous et al., 2015; Ortiz-Orendain et al., 2017), potentially removing large numbers of patients, disregarding additive effects, or ignoring those at highest risk of adverse effects. Another method used to summarize class exposures is the World Health Organization (WHO) defined daily dose (DDD) system (World Health Organization, 2019). A DDD is the average adult maintenance dose for the “main indication” of the drug. For example, to provide a summary measure of antipsychotic exposure, each antipsychotic’s prescribed daily dose would be divided by its respective DDD, and results for all antipsychotics would then be added together for each patient (Dubrall et al., 2020). However, DDDs were developed to study consumption trends and are derived from population average doses rather than pharmacologic potencies (World Health Organization, 2019), making DDD conversions less meaningful in drug effects studies.

An underutilized technique to summarize class exposures is direct dose conversion, a pharmacologically-based method which standardizes exposures to units of a common referent agent using dose equivalents. Once converted to common units, exposures can be easily summarized to assess drug effects and utilization. For example, Woods et al. aggregated chlorpromazine dose equivalents for atypical antipsychotics based on minimum effective doses for schizophrenia (Woods, 2003). Unfortunately, dose conversion methods are often avoided due to their complexity, and to our knowledge, few studies have implemented this method or provided tutorials for real-world drug research (Huybrechts et al., 2012a; Huybrechts et al., 2012b). We have previously applied direct dose equivalent methods to examine antipsychotic utilization and effects in Ontario (Tadrous et al., 2015; Mast et al., 2016). However, the focus of these studies was on results, and we were not able to describe the dose conversion method used in detail. As such, we believe a more in-depth example and tutorial is needed to increase the appropriate uptake of dose equivalent methods in pharmacoepidemiologic studies and to highlight the potential benefits and limitations of these methods. Thus, the purpose of this study was to provide a detailed example of dose conversion methods by quantifying the burden of antipsychotic use among a cohort of older adults with dementia.

## Methods

As part of a quality improvement initiative to understand the burden of antipsychotic prescribing among adults with dementia, we conducted a population-based cohort study of patients with dementia aged 66 years and older residing in Ontario, Canada who initiated antipsychotic drug therapy between January 1, 2009 and December 31, 2012. Linked Ontario administrative healthcare databases housed at ICES were used for cohort identification and prescription drug utilization (ICES, 2020). All Ontario residents receive healthcare coverage for medically necessary procedures and services; in addition, prescription medications listed on the Ontario Drug Benefits formulary are covered for patients over 65 years of age (Martin et al., 2018). The Canadian Institute for Health Information Discharge Abstract Database was used to obtain hospitalization records and the Ontario Health Insurance Plan database was used to identify diagnosis codes at physician visits. To obtain prescription data, the Ontario Drug Benefit (ODB) database was used, which contains claims information (drug, dose, administration route, day’s supply, and claim amount) for prescriptions dispensed to all Ontario residents over 65 years of age. These datasets were linked using unique, encoded identifiers and analyzed at ICES.

We identified the first claim for an antipsychotic for each person within the ODB database and defined the date of this prescription as their cohort entry date. The cohort was then restricted to patients with dementia using a validated case definition (Rochon, 2007) and the following patients were excluded: patients less than 66 years of age at cohort entry (to ensure that all patients had at least 1 year of prescription medication history), those prescribed any antipsychotic in the year prior to cohort entry, and those without any subsequent antipsychotic prescriptions in the 12 months after cohort entry. Patients were followed from cohort entry to death or the end of the study (12 months), whichever came first.

First, the antipsychotic dispensing data was cleaned using information from the day’s supply field, recommended prescribing intervals, time between prescription claim dates, and drug unit costs. Detailed data cleaning steps are provided in the Supplementary Material and may differ by jurisdiction. General rules for data cleaning were as follows: for oral and rectally administered medications, we obtained the date of the next prescription with the same medication name, if available. If this next prescription date was within 100 days, we kept the original day’s supply value. However, because dosing regimens and adherence to oral antipsychotic therapies can vary widely, if no subsequent prescription for the same medication was identified or if the subsequent prescription was dispensed more than 100 days later, we took a conservative approach and set the day’s supply value to the quantity dispensed divided by 5 (the maximum dosing interval for any antipsychotic therapy). For long-acting injectables, we kept the day’s supply as the original value if it was longer than the minimum dosing interval (e.g., 28 days for haloperidol decanoate), changed it to the minimum dosing interval if there was no subsequent long-acting antipsychotic prescription, or changed it to the duration between prescriptions if there was a subsequent long-acting prescription administered earlier than the minimum dosing interval. For short-acting injectables, we cleaned the quantity field by using information on the billable cost per mL and the total billed cost of the prescription. Then, we cleaned the day’s supply field using similar rules as those for oral medications.

The outcome of interest was the total chlorpromazine equivalent daily dose at cohort entry (first prescription) and at 6 and 12 months after initiation. At cohort entry, antipsychotic medication prescriptions dispensed on the first claim date were eligible, as no included patients had antipsychotic use in the year prior to first prescription. At 6 and 12 months after cohort entry, a 100-days lookback window was used to identify antipsychotic prescription dispensations with a day’s supply that covered each respective follow-up timepoint. Prescriptions outside of these 100-days lookback windows were not measured. The daily doses of prescriptions that overlapped with a follow-up timepoint were multiplied by the chlorpromazine dose equivalent, derived from pharmacologic studies on relative potency for effectiveness when available and clinical consensus for all others (Supplementary Table S1) (Woods, 2003; Gardner et al., 2010). Dose equivalents from all eligible prescriptions at each follow-up time point, regardless of drug or administration route, were summed to obtain a total chlorpromazine equivalent daily dose in milligrams. Injectable antipsychotic medications day’s supply cleaning rules and daily-dose equivalents were dependent on whether the medication was long-acting or short-acting. We calculated the median total equivalent daily dose at each time point among patients who remained on antipsychotic therapy, and we estimated the proportion of patients whose total equivalent daily dose was modified by data cleaning. Results were stratified by administration route. An example data cleaning and chlorpromazine daily-dose equivalent calculation is provided in Supplementary Table S2. This study was approved by the Research Ethics Board of Sunnybrook Health Sciences Centre, Toronto, ON, Canada.

## Results

**FIGURE 1:**
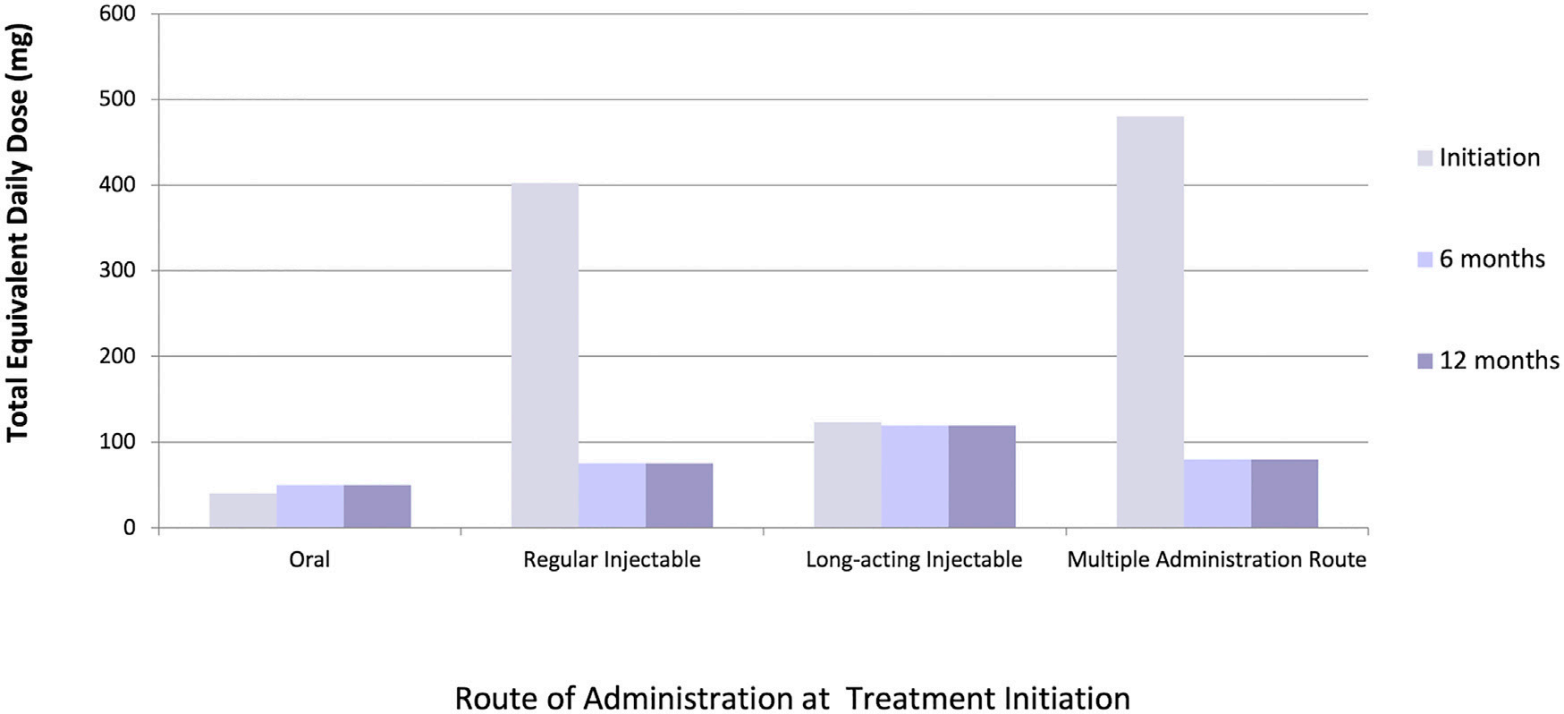
Median total chlorpromazine equivalent daily-dose (mg) at initiation (*n* = 45,442), 6 months (*n* = 27,387), and 12 months (*n* = 21,629) among various administration routes.

**FIGURE 2:**
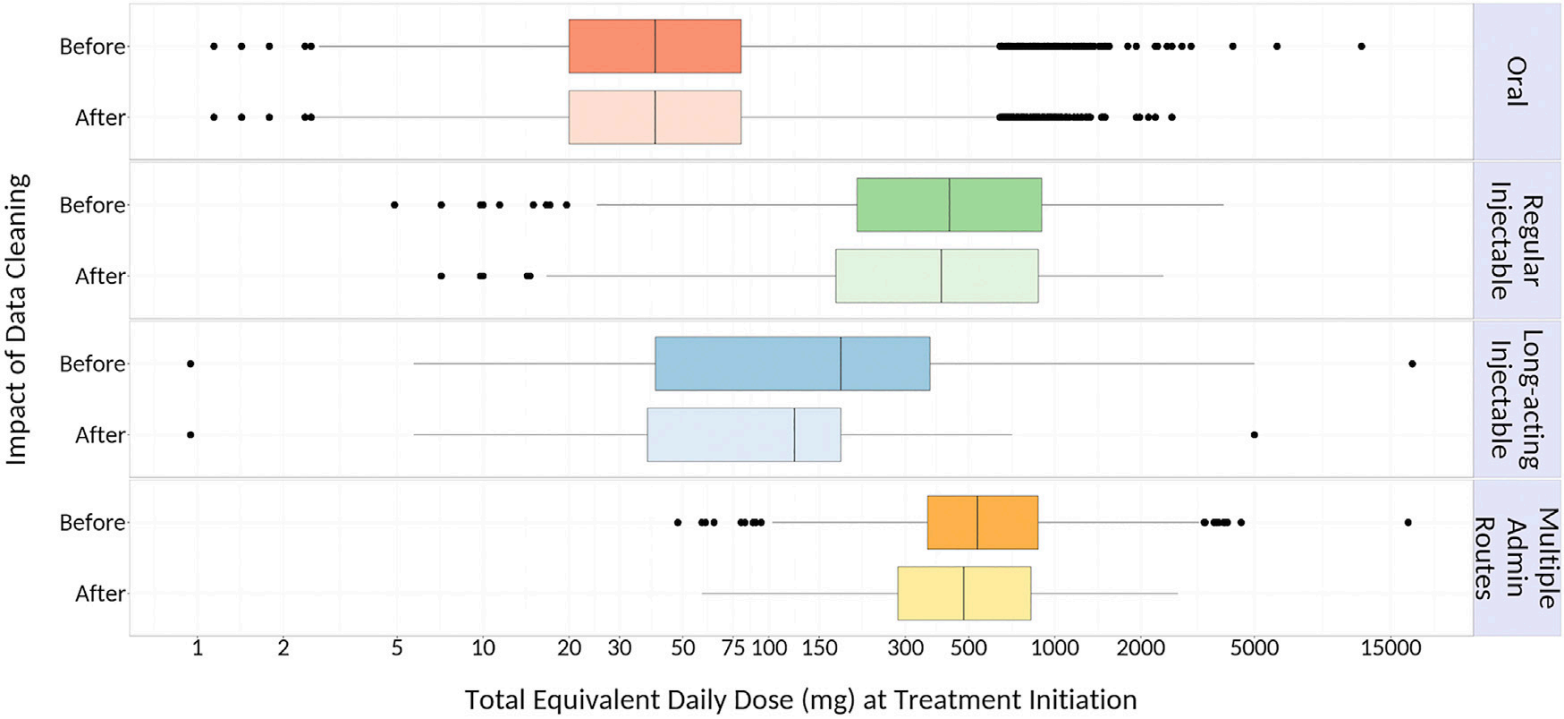
Impact of Data Cleaning on Calculated Total Equivalent Daily Dose at Initiation. {Outer edges of the boxes represent the 25th (Q1) and 75th (Q3) percentile values, respectively; middle line within each box represents the median (50th percentile value); dots represent outliers [values that are more than 1.5 times the interquartile range (IQR; Q3 – Q1) less than Q1 or more than 1.5*IQR greater than Q3], and whiskers extend to the smallest and largest values within this range.}

We identified 45,442 eligible older adults with dementia initiating antipsychotic medications. Of these, 27,387 and 21,629 patients remained on antipsychotic therapy at 6 and 12 months after initiation, respectively. The majority (97%) initiated oral therapy, followed by regular injectables (2%), multiple administration routes (1%) and long-acting injectables (0.5%); eighty-one percent of patients initiated an atypical (versus typical) antipsychotic medication. At initiation, 14% of patients received multiple antipsychotic drug prescriptions simultaneously, compared to 22% at 6 months and 16% at 12 months after initiation. Median equivalent daily doses differed across administration routes, with the highest initial doses seen among patients receiving regular injectable and multiple administration routes; however, dose equivalents in these groups decreased over time to reach doses that were similar to other administration routes by 6 months of follow-up (Figure 1). Data cleaning modified 3, 16, 36, and 42% of total equivalent daily-doses in patients initiating oral, regular injectable, long-acting injectable, and multiple administration routes, respectively (Figure 2), with most modified doses decreased after cleaning.

## Discussion

In a cohort of 45,442 older adults with dementia in Ontario initiating antipsychotic therapy, a data cleaning and dose conversion algorithm was applied to quantify exposures across the antipsychotic drug class and illustrate a detailed, real-world example of direct dose conversion methods. Dose conversion was a feasible and efficient method to compare antipsychotic exposures between administration routes and time points. Results identified that patients initiated on regular injectable therapies and multiple administration routes received higher equivalent daily-doses than those initiated on long-acting injectables and oral therapies. Because daily-doses at follow-up timepoints could be calculated from multiple overlapping prescriptions but baseline doses were from a single prescription, this finding is unexpected. However, we hypothesize that some patients with dementia may have been initiated on high daily-doses of antipsychotics to control agitation until other medications can take effect to manage BPSD symptoms, with subsequent doses decreased or tapered if the patient’s symptoms improved.

Next, data cleaning had a substantial impact on total equivalent daily-doses, particularly among patients receiving long-acting injectables and multiple administration routes. This disparity may have arisen from errors in the day’s supply field for injectable medications (e.g., day’s supply value of 1 for a 28-days one-time injection that would result in a higher original daily dose), which have been previously reported for claims for injectable osteoporosis prescriptions in Ontario (Burden et al., 2013). Fewer errors for oral therapies were seen, potentially because day’s supplies for oral medications are often identical to the quantity dispensed. Finally, this study also suggests that excluding patients on multiple therapies can substantially reduce the study population size, as 14% of patients were initiated on multiple antipsychotics and this proportion increased throughout the first year.

This algorithm has been successfully applied to study antipsychotic utilization and drug effects in Ontario, yet has not been described in detail so that others may utilize it until now (Tadrous et al., 2015; Mast et al., 2016). Mast et al. found that older adults with dementia initiated on higher chlorpromazine equivalent daily-doses of antipsychotics discontinued more rapidly than those initiated on lower doses, showing effect modification of persistence by dose (Mast et al., 2016). Further, a 2015 report on the use of antipsychotics in older adults in Canada found differences in equivalent doses between patients residing in the community versus nursing homes and between those initiating typical versus atypical antipsychotics (Tadrous et al., 2015). Similar dose-conversion methods appear to have been employed in real-world studies in other jurisdictions to examine antipsychotic persistence (Iwata et al., 2020), dose-initiation (Takahashi et al., 2020), risk of death (Huybrechts et al., 2012b), and potential effect modification of adverse events (Huybrechts et al., 2012a).

Dose conversion can be applied in pharmacoepidemiologic studies of other drug-classes with 1) many different drugs, 2) a variety of formulations, or 3) potential for concurrent in-class exposures. We recommend that if 2 or more of these conditions are met for a drug class, investigators should consider dose conversion to standardize exposures. Opioids and benzodiazepines are drug classes which meet all three criteria and for which dose conversion has proven beneficial. For example, Gomes et al. found a dose-response relationship between morphine equivalent daily-doses of opioids and fatal opioid overdose in patients in Ontario (Gomes et al., 2011), and Brandt et al. reported that diazepam milligram equivalent conversion allowed more advanced interpretation of benzodiazepine use in Canada versus other standardization methods (Brandt et al., 2019).

Although antipsychotic DDDs have been used in pharmacoepidemiologic applications, we believe that DDD methods are less ideal than dose conversion primarily because DDDs are not based on pharmacologic activity (World Health Organization, 2019). For example, when different antipsychotic dose standardization methods were compared to determine how well they predicted extrapyramidal symptoms (EPS), a dose-related adverse effect, only chlorpromazine-equivalent doses were significantly different between patients with and without EPS (Barr et al., 2010). DDD-based doses were similar between groups. These findings suggest that antipsychotic dose equivalents may be more closely related to pharmacologic activity than DDDs and thus more appropriate in pharmacoepidemiologic studies.

There are limitations to this algorithm and dose conversion in general. Firstly, our rule-based data cleaning did rely in part on day’s supply information, which may not be available in some dispensing data. However, our algorithm also incorporated rules based on cost lists and dates between prescriptions to quantify exposures, illustrating that jurisdiction- and disease-specific knowledge may be a feasible alternative to using day’s supply in data cleaning. Moreover, even when the actual dosage of drug is accurately measured through prescription claims, actual consumption may not be equivalent to what is measured due to low adherence, particularly for oral medications. Secondly, dose conversion relies on validated dose equivalents, established from pharmacodynamic studies (Woods, 2003) or clinical consensus (Gardner et al., 2010), which are not always available, established via transparent methods, or valid when co-medications alter antipsychotic metabolism. Moreover, consensus dose equivalents often lag for newer drugs. Another major limitation of dose conversion methods is that they only consider the equivalence of one mechanism–for example, dopamine receptor activity of antipsychotics in the present study; however, drugs within the same class may have differing activity for other pharmacologic effects (e.g., cholinergic activity) that may modify effects beyond one mechanism. This limitation may be especially important when examining the effectiveness of medications; dose conversion methods may be more appropriate for studies of adverse effects, off-label uses, or utilization studies.

Dose conversion can be successfully applied after data cleaning to standardize antipsychotic drug exposures in the study of real-world drug safety and effectiveness. With adequate knowledge of their strengths and limitations, conversion methods can be considered for drug effects studies of antipsychotics and other medication classes with similar complexities, such as opioids and benzodiazepines.

## Data Availability

The data analyzed in this study is subject to the following licenses/restrictions: The dataset from this study is held securely in coded form at ICES. While data sharing agreements prohibit ICES from making the dataset publicly available, access may be granted to those who meet pre-specified criteria for confidential access, available at www.ices.on.ca/DAS. The full dataset creation plan and underlying analytic code are available from the authors upon request, understanding that the computer programs may rely upon coding templates or macros that are unique to ICES and are therefore either inaccessible or may require modification. Requests to access these datasets should be directed to www.ices.on.ca/DAS.

## References

[B1] Barr A. M. Honer W. G. Johnson J. L. Wu T. K. Y. Procyshyn R. M. (2010). A Comparison of Antipsychotic Drug-Defined Daily Doses versus Chlorpromazine Equivalent Doses in Patients with or without Extrapyramidal Motor Symptoms. J. Clin. Psychopharmacol. 30 (6), 741–743. 10.1097/JCP.0b013e3181fab7ca 21057241

[B2] Brandt J. Alessi-Severini S. Singer A. Leong C. (2019). Novel Measures of Benzodiazepine & Z-Drug Utilisation Trends in a Canadian Provincial Adult Population (2001-2016 ). J. Popl Ther. Clin. Pharmacol. 26 (1), e22–e38. 10.22374/1710-6222.26.1.3 31002486

[B3] Burden A. M. Huang A. Tadrous M. Cadarette S. M. (2013). Variation in the Days Supply Field for Osteoporosis Medications in Ontario. Arch. Osteoporos. 8 (1-2). 128. 10.1007/s11657-013-0128-1 23475734

[B4] Dubrall D. Just K. S. Schmid M. Stingl J. C. Sachs B. (2020). Adverse Drug Reactions in Older Adults: a Retrospective Comparative Analysis of Spontaneous Reports to the German Federal Institute for Drugs and Medical Devices. BMC Pharmacol. Toxicol. 21 (1), 25. 10.1186/s40360-020-0392-9 32293547 PMC7092423

[B5] Gardner D. M. Murphy A. L. O'Donnell H. Centorrino F. Baldessarini R. J. Baldessarini R. J. (2010). International Consensus Study of Antipsychotic Dosing. Am. J. Psychiatry 167, 686–693. 10.1176/appi.ajp.2009.09060802 20360319

[B6] Gill S. S. Bronskill S. E. Normand S.-L. T. Anderson G. M. Sykora K. Lam K. (2007). Antipsychotic Drug Use and Mortality in Older Adults with Dementia. Ann. Intern. Med. 146 (11), 775. 10.7326/0003-4819-146-11-200706050-00006 17548409

[B7] Gomes T. Mamdani M. M. Dhalla I. A. Paterson J. M. Juurlink D. N. (2011). Opioid Dose and Drug-Related Mortality in Patients with Nonmalignant Pain. Arch. Intern. Med. 171 (7). 686-91. 10.1001/archinternmed.2011.117 21482846

[B8] Huybrechts K. F. Gerhard T. Crystal S. Olfson M. Avorn J. Levin R. (2012). Differential Risk of Death in Older Residents in Nursing Homes Prescribed Specific Antipsychotic Drugs: Population Based Cohort Study. BMJ 344 (feb23 2), e977. 10.1136/bmj.e977 22362541 PMC3285717

[B9] Huybrechts K. F. Schneeweiss S. Gerhard T. Olfson M. Avorn J. Levin R. (2012). Comparative Safety of Antipsychotic Medications in Nursing home Residents. J. Am. Geriatr. Soc. 60 (3), 420–429. 10.1111/j.1532-5415.2011.03853.x 22329464 PMC3302976

[B10] ICES (2020). Data Available through DAS. Available at: https://www.ices.on.ca/DAS/Data (Accessed June 26, 2020).

[B11] Iwata N. Inagaki A. Sano H. Niidome K. Kojima Y. Yamada S. (2020). Treatment Persistence between Long-Acting Injectable versus Orally Administered Aripiprazole Among Patients with Schizophrenia in a Real-World Clinical Setting in Japan. Adv. Ther. 37 (7), 3324–3336. 10.1007/s12325-020-01396-w 32500455 PMC7314731

[B12] Martin D. Miller A. P. Quesnel-Vallée A. Caron N. R. Vissandjée B. Marchildon G. P. (2018). Canada's Universal Health-Care System: Achieving its Potential. The Lancet 391 (10131), 1718–1735. 10.1016/S0140-6736(18)30181-8 PMC7138369 29483027

[B13] Mast G. Fernandes K. Tadrous M. Martins D. Herrmann N. Gomes T. (2016). Persistence of Antipsychotic Treatment in Elderly Dementia Patients: A Retrospective, Population-Based Cohort Study. Drugs - Real World Outcomes 3 (2), 175–182. 10.1007/s40801-016-0073-6 27398296 PMC4914533

[B14] Ortiz-Orendain J. Castiello-de Obeso S. Colunga-Lozano L. E. Hu Y. Maayan N. Adams C. E. (2017), Antipsychotic Combinations for Schizophrenia Cochrane Schizophrenia Group. Cochrane Database Syst Rev. Published online June 28. 10.1002/14651858.CD009005.pub2 PMC6481822 28658515

[B15] Rochon P. A. (2007). Variation in Nursing Home Antipsychotic Prescribing Rates. Arch. Intern. Med. 167 (7), 676. 10.1001/archinte.167.7.676 17420426

[B16] Schneider L. S. Dagerman K. Insel P. S. (2006). Efficacy and Adverse Effects of Atypical Antipsychotics for Dementia: Meta-Analysis of Randomized, Placebo-Controlled Trials. Am. J. Geriatr. Psychiatry 14 (3), 191–210. 10.1097/01.JGP.0000200589.01396.6d 16505124

[B17] Siafis S. Tzachanis D. Samara M. Papazisis G. (2018). Antipsychotic Drugs: From Receptor-Binding Profiles to Metabolic Side Effects. Cn 16 (8), 1210–1223. 10.2174/1570159X15666170630163616 PMC6187748 28676017

[B18] Sultana J. Calabró M. Garcia-Serna R. Ferrajolo C. Crisafulli C. Mestres J. (2017). Biological Substantiation of Antipsychotic-Associated Pneumonia: Systematic Literature Review and Computational Analyses. PLoS One 12 (10), e0187034, 10.1371/journal.pone.0187034 29077727 PMC5659779

[B19] Tadrous M. Martins D. Herrmann N. Fernandes K. Yao Z. Singh S. (2015). Antipsychotics in the Elderly. Final Report: Pharmacoepidemiology Unit. Ontario Drug Policy Research Network (ODPRN). Available at: https://odprn.ca/wp-content/uploads/2015/06/Antipsychotic-Pepi-Report.pdf (Accessed July 26, 2021). Published

[B20] Takahashi T. Otsubo T. Kunisawa S. Sasaki N. Imanaka Y. (2020). Factors Associated with High‐dose Antipsychotic Prescriptions in Outpatients with Schizophrenia: An Analysis of Claims Data from a Japanese Prefecture. Neuropsychopharmacol. Rep. 40, 224–231. Published online May 26. 10.1002/npr2.12109 32452649 PMC7722669

[B21] Woods S. W. (2003). Chlorpromazine Equivalent Doses for the Newer Atypical Antipsychotics. J. Clin. Psychiatry 64 (6), 663–667. 10.4088/JCP.v64n0607 12823080

[B22] World Health Organization (2019). ATC/DDD Index. Available at: https://www.whocc.no/atc_ddd_index/. Published online December 13, 2018. (Accessed May 22, 2019).

