# Supplementary material for: Greater than the Sum: Applying Daily-Dose Equivalents to Antipsychotic Prescription Claims to Study Real-World Effects
Source: Front Pharmacol. 2021 Aug 6;12:709349. doi: 10.3389/fphar.2021.709349 (PMC8378135; doi:10.3389/fphar.2021.709349)
Supplement: Supplementary file 1 [file DataSheet1.docx]

**Supplementary Material 1. Data cleaning algorithm used with Ontario Drug Benefit (ODB) claims data to calculate chlorpromazine equivalent daily doses (eDD).**

**Formula**: eDD=(quantity*strength/days’ supply)*CZDeq

*Where eDD = chlorpromazine equivalent daily dose and CZDeq is the chlorpromazine dose equivalent adapted from Gardner* et al. and *Woods* et al. *(Supplementary Table 1).*^1,2^

**Notes:**

1. **For capsules, tablets, and rectal:**
   1. use quantity as provided
   2. For days’ supply: if days’ supply is
      1. < quantity/5, look for next prescription with same drug name.
         1. If found:
            1. If next prescription date is > 100 days, then set days’ supply to quantity/5.
            2. Otherwise, set days’ supply to max (quantity/5, difference in prescription dates).
         2. If not found, set days’ supply to quantity/5.
      2. ≥ quantity/5, then use days’ supply
2. **For long acting injectable:**
   1. use quantity as provided
   2. For days’ supply: If days’ supply is:
      1. < minimum limit of the dosing interval for that drug:
         1. Look for next long-acting prescription (not necessarily for the same drug).
            1. If no subsequent long acting prescription, then set days’ supply to maximum limit of the dosing interval for that drug.
            2. If subsequent long-acting prescription, then set days’ supply to lesser of the following values: days between in prescription dates or maximum limit of the dosing interval for that drug.
      2. ≥ minimum limit of the dosing interval for that drug, then use the days’ supply
3. **For regular injectables and oral/liquids:**
   1. use cleaned quantity variable:
      1. Link the index prescription record to unit cost per ml based on the index date year and the din to retrieve the cost per ml
      2. Calculate new quantity as:
         1. If Cost/(cost/ml) < 1 then new quantity=cost/(cost/mL)
         2. Otherwise new quantity=cost/(cost/mL), rounded to the nearest 1 mL
   2. For days’ supply: if days’ supply is
      1. < quantity/5, look for next prescription with same drug name.
         1. If found:
            1. If next prescription date is > 100 days, set days’ supply to quantity/5
            2. Otherwise, set days’ supply to the greater of the following values: quantity/5 or days between prescription dates
         2. If not found, set days’ supply to quantity/5
      2. ≥ quantity/5, then use days’ supply

| **Supplementary Table 1. Chlorpromazine equivalents used for Calculating Daily Dosing**^1,2^ | | |
| --- | --- | --- |
| Class | Drug | Dose Equivalent Factor |
| Atypical | Aripiprazole | 20 |
|  | Asenapine | 20 |
|  | Clozapine | 1.50 |
|  | Olanzapine | 30 |
|  | Lurasidone | 5 |
|  | Paliperidone | 66.6 |
|  | Quetiapine | 0.8 |
|  | Risperidone | 100 |
|  | Ziprasidone | 3.75 |
| Typical | Chlorpromazine | 1 |
|  | Flupenthixol | 60 |
|  | Fluphenazine | 50 (Long-acting inj = 17.8 or 14.2) |
|  | Haloperidol | 60 (long-acting inj = 35.7) |
|  | Loxapine | 10 |
|  | Methotrimeprazine | 2 |
|  | Molindone | 6 |
|  | Periciazine | 12 |
|  | Perphenazine | 20 |
|  | Pimozide | 75 |
|  | Pipotiazine | 2 |
|  | Prochlorperazine | 6.86 |
|  | Thioridazine | 1.20 |
|  | Thiothixene | 20 |
|  | Trifluperazine | 30 |
|  | Zuclopenthixol | 12 (long-acting inj = 4.3) |

**References:**

1. Gardner DM, Murphy AL, O’Donnell H, Pharm B, Centorrino F, Baldessarini RJ. International Consensus Study of Antipsychotic Dosing. *Am J Psychiatry*. 2010;167(6):686-693.

2. Woods SW. Chlorpromazine Equivalent Doses for the Newer Atypical Antipsychotics. *J Clin Psychiatry*. 2003;64(6):663-667. doi:10.4088/JCP.v64n0607

**Supplementary Table 2. Example of Data Cleaning and Dose Conversion.**

| **Step** | **Input Data** | **Action** | **Output Data** |
| --- | --- | --- | --- |
| Data Cleaning (1) | *Baseline Prescription:*  QUETIAPINE (SEROQUEL XR) 50 mg  ROUTE: ORAL  QUANTITY: 60  DAYS SUPPLY: 3  DATE: 01/01/2010 | a. Assess type of prescription   - This is an oral tablet, so follow data cleaning instructions for “**capsules, tablets, and rectal”** | N/A |
| Data Cleaning (2) | *Baseline Prescription:*  QUETIAPINE (SEROQUEL XR) 50 mg  ROUTE: ORAL  QUANTITY: 60  DAYS SUPPLY: 3  DATE: 01/01/2010 | a. Leave quantity as-is.  b. Clean days’ supply using instructions   - Days supply (=3) is less than quantity/5 (60/3=20) - Look for next prescription with same drug name | N/A |
| Data Cleaning (3) | *Baseline Prescription:*  QUETIAPINE (SEROQUEL XR) 50 mg  ROUTE: ORAL  QUANTITY: 60  DAYS SUPPLY: 3  DATE: 01/01/2010  *Next Prescription:*  QUETIAPINE (SEROQUEL XR) 50 mg  ROUTE: ORAL  QUANTITY: 60  DAYS SUPPLY: 30  **DATE: 01/31/2010** | a. Assess number of days between original prescription and next prescription   - Number of days: 30   b. Because number of days between prescriptions is < 100, set days’ supply value to maximum of: quantity/5 (=12) OR number of days between prescriptions (=30) | *Baseline Prescription:*  QUETIAPINE (SEROQUEL XR) 50 mg  ROUTE: ORAL  QUANTITY: 60  ORIGINAL DAYS SUPPLY: 3  **UPDATED DAYS SUPPLY: 30**  DATE: 01/01/2010 |
| Dose Conversion (1) | *Baseline Prescription:*  QUETIAPINE (SEROQUEL XR) 50 mg  ROUTE: ORAL  QUANTITY: 60  ORIGINAL DAYS SUPPLY: 3  UPDATED DAYS SUPPLY: 30  DATE: 01/01/2010 | a. Multiply milligrams of original drug (quetiapine) by quantity supply to obtain total amount of quetiapine exposure from this prescription (50 mg x 60 = 3,000 mg)  b. Divide total exposure from this prescription by days supply to calculate daily dose (3,000 mg/30 days= 100 mg/day quetiapine) | *Baseline Prescription:*  QUETIAPINE (SEROQUEL XR) 50 mg  ROUTE: ORAL  QUANTITY: 60  ORIGINAL DAYS SUPPLY: 3  UPDATED DAYS SUPPLY: 30  DATE: 01/01/2010  **DAILY DOSE OF ORIGINAL DRUG: 100 MG** |
| Dose Conversion (2) | *Baseline Prescription:*  QUETIAPINE (SEROQUEL XR) 50 mg  ROUTE: ORAL  QUANTITY: 30  ORIGINAL DAYS SUPPLY: 3  UPDATED DAYS SUPPLY: 30  DATE: 01/01/2010  **DAILY DOSE OF ORIGINAL DRUG: 100 MG** | Multiply amount of original drug by chlorpromazine dose equivalent from Supplementary Table 1 (100 mg/day x 0.8 = 80 chlorpromazine equivalent mg)  a. If index prescription, stop. If prescription is within 6-month or 12-month ascertainment window, repeat data cleaning with all other eligible prescriptions in window (any drug and any administration route), and sum chlorpromazine dose equivalents for that window to obtain total exposure at follow-up time point. | *Baseline Prescription:*  QUETIAPINE (SEROQUEL XR) 50 mg  ROUTE: ORAL  QUANTITY: 60  ORIGINAL DAYS SUPPLY: 3  UPDATED DAYS SUPPLY: 30  DATE: 01/01/2010  DAILY DOSE OF ORIGINAL DRUG: 100 MG  **EQUIVALENT CHLORPROMAZINE DAILY DOSE (MG): 80 MG** |
